# Identification of two mutations of the *RHO* gene in two Chinese families with retinitis pigmentosa: Correlation between genotype and phenotype

**Published:** 2012-12-14

**Authors:** Zhe Pan, Tingting Lu, Xiaohui Zhang, Hanjun Dai, Weiyu Yan, Fengge Bai, Yang Li

**Affiliations:** Beijing Institute of Ophthalmology, Beijing Tongren Eye Center, Beijing Tongren Hospital, Capital Medical University, Beijing Ophthalmology and Visual Sciences Key Laboratory, Beijing, China

## Abstract

**Purpose:**

To describe the clinical and genetic findings in two Chinese families with retinitis pigmentosa (RP).

**Methods:**

Two unrelated families were examined clinically. After informed consent was obtained, genomic DNA was extracted from the venous blood of all participants. Genotyping and haplotyping analysis was performed on the known genetic loci for autosomal dominant retinitis pigmentosa (adRP) with a panel of polymorphic markers in the two families, and then mutation screening of all coding exons of the *RHO* gene was performed by direct sequencing of PCR-amplified DNA fragments. Whenever substitutions were identified in a patient, restriction fragment length polymorphism analysis was performed on all available family members and on 100 normal controls.

**Results:**

Clinical examination and pedigree analysis revealed two four-generation families (83 and 112) with adRP. A significant two-point linkage odd disequilibrium (LOD) score was generated at marker D3S1292 (Zmax=1.90, θ=0) for family 83 and (Zmax=2.77, θ=0) for family 112, respectively, and further linkage and haplotype studies confined the disease locus to 3q21–22 where the *RHO* gene is located. Mutation screening of the *RHO* gene in the two families revealed a G→C transversion at position 505 (p.A169P) of the cDNA sequence in family 83 and a C→A transversion at position 1040 (p.P347Q) of the cDNA in family 112. The novel p.A169P and recurrent p.P347Q mutations cosegregated with the phenotypes of the two families. Secondary structure prediction suggested that the mutant rhodopsin 169P led to significant secondary structure changes between residues 165 and 169, which may interfere with the correct folding of the transmembrane domain.

**Conclusions:**

Two mutations of the *RHO* gene were identified in two Chinese families with adRP. Our findings further suggest codon 347 is the mutation hotspot of the *RHO*.

## Introduction

Retinitis pigmentosa (RP) is a clinically and genetically heterogeneous group of retinal dystrophies, characterized by progressive degeneration of the photoreceptors. Clinical features include progressive night blindness, constriction and gradual loss of the peripheral visual field, and eventual loss of visual acuity. With an incidence of 1 in 4,000 people, RP can be inherited as an autosomal recessive (arRP), an autosomal dominant (adRP), or an X-linked recessive (xlRP) pattern [1,2]. To date, at least 23 causative genes have been identified for adRP, 35 for arRP, and two for xlRP (RetNet).

The *RHO* gene, located on chromosome 3q21–22, was the first photoreceptor specific gene found to be mutated in adRP [3-5]. This gene encodes protein rhodopsin, the light-absorbing molecule that initiates the signal transduction cascade in rod photoreceptors. Rhodopsin, which has 348 amino acids, is organized into three distinct regions: cytoplasmic, transmembrane (TM), and intradiscal domains. The *RHO* gene is the most common gene implicated in adRP, and more than 120 different mutations have been identified in different sites of the gene, most of which are missense mutations (RetNet) [3-14].

Based on their biochemical and cellular properties, rhodopsin mutations in adRP have been classified into six groups, but most are grouped into class I or class II [15]. Class I mutations, which predominantly occur in the C-terminus of the protein, can fold normally, but are not correctly transported to the outer segment. Class II mutations, which cannot fold correctly, are retained in the endoplasmic reticulum (ER) and are unable to form a functional chromophore with 11-cis-retinal. Class II mutations usually occur in the intradiscal and transmembrane domains of rhodopsin.

In this study, we investigated two Chinese families with adRP. After linkage and haplotyping analysis, the disease-causing gene was mapped to the *RHO* region. Then mutation screening of the *RHO* gene was performed in the two adRP families. One novel mutation and one recurrent mutation were identified. We compared our findings to those of other studies of *RHO* mutations in the Chinese population.

## Methods

### Clinical data and sample collection

This study adhered to the tenets of the Declaration of Helsinki for research involving human subjects. The Beijing Tongren Hospital Joint Committee on Clinical Investigation approved the study. Two Chinese families with nonsyndromic RP were referred to Beijing Tongren Hospital. After informed consent was obtained, each participant underwent careful ophthalmologic examinations, including best-corrected visual acuity testing using E decimal charts, slit-lamp biomicroscopy, and fundus examination with dilated pupils. Some of the patients had visual field testing and electroretinogram (ERG) examination. Peripheral blood was obtained with venipuncture, and genomic DNA was extracted using Whole Blood DNA Extraction Kit (Vigorous Biotechnology, Beijing, China).

### Linkage and haplotyping analysis

Genotyping was performed with 41 microsatellite markers from autosomes for the known adRP loci in the two families (Appendix 1). Fine mapping primer sequences were obtained from the Human Genome Database (GDB). Linkage odd disequilibrium (LOD) scores were calculated for the markers with two-point linkage analysis using Linkage package 5.2. We modeled the disease as an autosomal dominant trait with 100% penetrance. Pedigree and haplotype maps were constructed using Cyrillic V. 2.0 software.

### Mutation screening of the *RHO* gene

Mutation screening was performed in the two families using direct DNA sequence analysis. The five coding regions and the exon-intron boundaries of the *RHO* gene were amplified with polymerase chain reaction (PCR) in the patients of the two families. The pairs of primers for five exons were used according to previously published article (Table 1) [12]. For direct sequencing, amplicons were purified (Shenneng Bocai PCR purification kit; Shenneng, Shanghai, China). An automatic fluorescence DNA sequencer (ABI, Prism 373A; Applied Biosystems Inc., Foster City, CA), used according to the manufacturer’s instructions, sequenced the purified PCR products in forward and reverse directions. Nucleotide sequences were compared with the published cDNA sequence of the *RHO* gene (GenBank NM_000539). For the *RHO* gene, cDNA numbering, +1 corresponds to A in the ATG translation initiation codon in rhodopsin.

**Table 1:** Primer information for the RHO gene sequence.

Primer	Forward sequence (5′-3′)	Reverse sequence (5′-3′)	Products (bp)	Tm (°C)
Exon1	AGCTCAGGCCTTCGCAGCAT	GAGGGCTTTGGATAACATTG	456	58
Exon2	GAGTGCACCCTCCTTAGGCA	TCCTGACTGGAGGACCCTAC	289	60
Exon3	CTGTTCCCAAGTCCCTCACA	CTGGACCCTCAGAGCCGTGA	260	58
Exon4	ATGCATCTGCGGCTCCTGCT	CCTGGGAGTAGCTTGTCCTT	358	60
Exon5	ACGTGCCAGTTCCAAGCACA	ATTCTGCACAGGCGCTGCTC	273	58

### Restriction fragment length polymorphism analysis

To confirm the variations found in the sequencing, restriction endonuclease AciI and StuI (New England Biolabs, Ipswich, MA) were used in all available family members and 100 normal controls, respectively. The reaction was performed in a 10 μl volume containing 9.5 μl PCR product and 0.5 μl enzyme (10 U/μl). The reaction was incubated overnight at 37 °C, after which the whole digest was run on a 1% agarose gel stained with ethidium bromide and visualized under ultraviolet light.

### Bioinformatics analysis

Garnier-Osguthorpe-Robson (GOR) software was used to predict the effect of the mutation on the secondary structure of RHO (Biotools) [16]. The Polymorphism Phenotyping (PolyPhen) program was used to predict the potential functional impact of an amino acid change [17].

## Results

### Clinical findings

**Figure 1:**
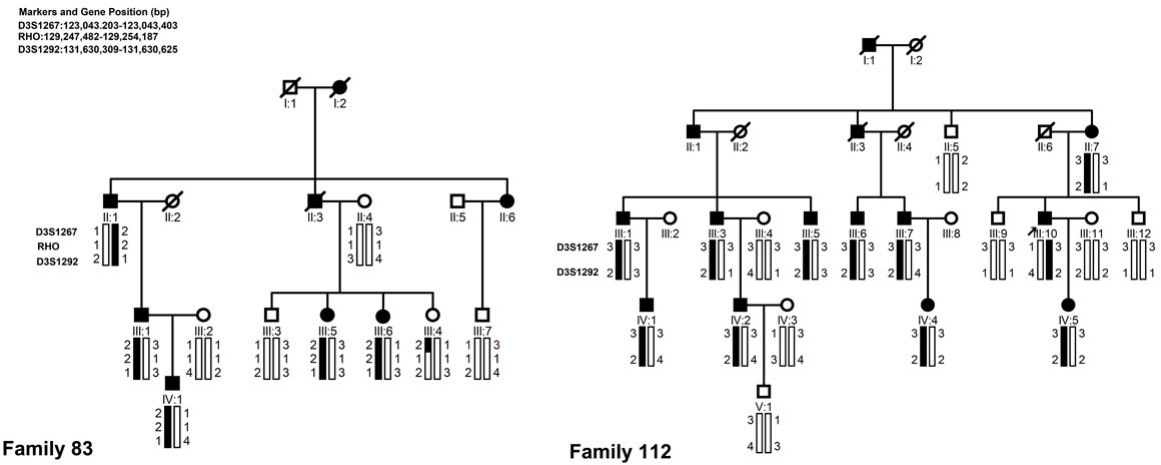
Family structure and haplotype analysis of two Chinese families with retinitis pigmentosa. Pedigree and haplotype analysis of the families with retinitis pigmentosa (RP) showed segregation with two microsatellite markers on chromosome 3 listed in descending order from the centromeric end. Squares indicate males; circles indicate females; slashed symbols indicate deceased; solid symbols indicate affected; open symbols indicate unaffected.

**Figure 2:**
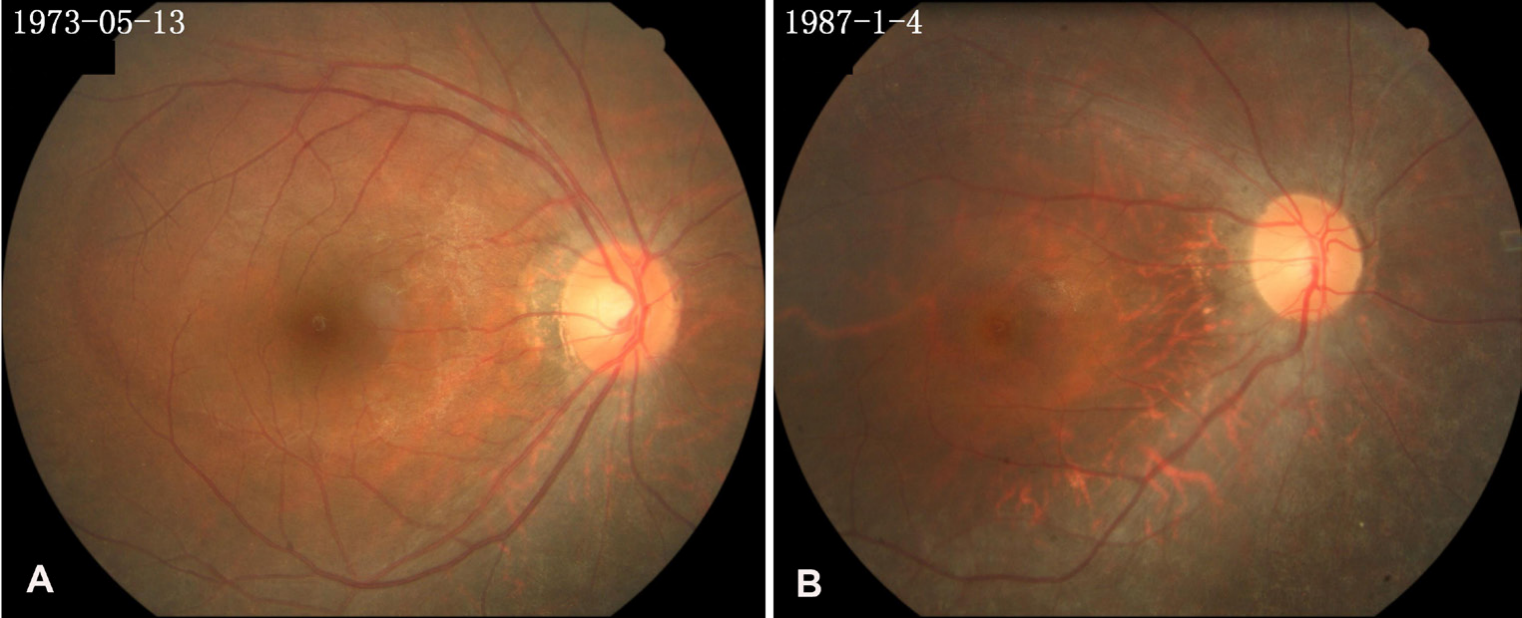
Fundus appearance of two patients with retinitis pigmentosa. **A**: Fundus appearance of the right eye of patient III:6 in family 83 shows mild atrophic retinal pigment epithelial changes and a few pigments in the inferior periphery fundus. **B**: Fundus appearance of the right eye of patient IV:5 in family 112 presents atrophic retinal pigment epithelial changes, attenuation of the retinal vessels, and irregular pigment clumps in the retina. Numbers in the upper-left corners are the patients’ birth dates.

This study identified two four-generation Chinese families diagnosed with non-syndromic RP. The inheritance pattern in the families was autosomal dominant (Figure 1). In family 83, most patients had experienced night blindness around age ten and exhibited characteristic RP fundus appearance, including atrophic retinal pigment epithelial changes with a great deal of bone spicule-like pigmentation. However, individual III:6 at age 38 years had vision of 1.2 in both eyes and did not have the night blindness complaint. Fundus examinations showed mild retinal pigment epithelial atrophy in the mid-periphery and a few bone spicules in the inferior periphery fundus (Figure 2). ERG testing revealed undetectable rod responses and an 80% reduction in cone responses. In family 112, all patients had experienced night blindness in their early childhood and had attenuation of the retinal vessels and bone spicule-like pigmentation in their fundi (Figure 2). ERG testing of the proband and his daughter showed undetectable rod and cone responses. Two individuals (III:6 and III:10) were diagnosed with angle closure glaucoma due to their intraocular pressure being elevated and the anterior angle closed. Detailed clinical information for each family’s affected members is summarized in Table 2.

**Table 2:** The clinical features of patients from the two families

Family number	Patient number	Age	Onset age of night blindness	Best corrected vision (OD/OS)	Cataract	Fundus appearance*	Visual field	Glaucoma
83	II-1	78	C	LP/LP	YES	NA	NA	NO
	III-1	58	C	0.5/0.6	YES	YES	Constriction, central 20 degrees	NO
	IV-1	30	C	1.0/1.0	NO	YES	Constriction, central 30 degrees	NO
	III-5	44	C	0.8/0.7	NO	YES	Constriction, central 30 degrees	NO
	III-6	38	NO	1.2/1.2	NO	only in inferior quadrant	Mild constriction	NO
112	III-1	74	EC	NLP	YES	NA	NA	NO
	IV-1	43	EC	0.3/0.5	YES	YES	NA	NO
	III-3	67	EC	0.05/0.05	YES	NA	NA	NO
	IV-2	40	EC	0.5/0.5	YES	YES	NA	NO
	III-5	63	EC	0.1/0.1	YES	YES	NA	NO
	III-6	57	EC	HM	YES	YES	NA	YES
	III-7	49	EC	0.1/0.1	YES	YES	NA	NO
	IV-4	20	EC	0.5/0.5	NO	YES	NA	NO
	II-7	87	EC	NLP	YES	YES	NA	NO
	III-10	48	EC	0.6/0.01	IOL/ YES	YES	Constriction, central 10 degrees	YES
	IV-5	25	EC	1.0/1.0	NO	YES	Constriction, central 30 degrees	NO

Abbreviations: C, childhood; EC, early childhood; OD, right eye; OS, left eye; HM, hand movement; NLP, no light perception; LP, light perception; NA, not available, *intraretinal bone spicule pigments in four quadrants.

### Genotyping results

Two families were genotyped with 41 polymorphic markers around the known adRP loci. The mapping results excluded the other known adRP loci with the exception of the *RHO* gene. The marker results for D3S1292 and D3S1267 were fully informative for linkage for the two families. For family 83, the two-point LOD scores for D3S1292 and D3S1267 with 100% penetrance were 1.90 (θ=0) and 1.81(θ=0), respectively. For family 112, the two-point LOD scores for D3S1292 and D3S1267 were 2.77 (θ=0) and 2.44 (θ=0), respectively. There were no affected recombinants in either of the two families (Figure 1). Although a meiotic breakpoint was observed in an unaffected family member (III:4) of family 83, marker D3S1292 was close to the *RHO* locus whereas marker D3S1267 was further centromeric and did not comprise any part of the *RHO* gene.

### Mutation analysis

**Figure 3:**
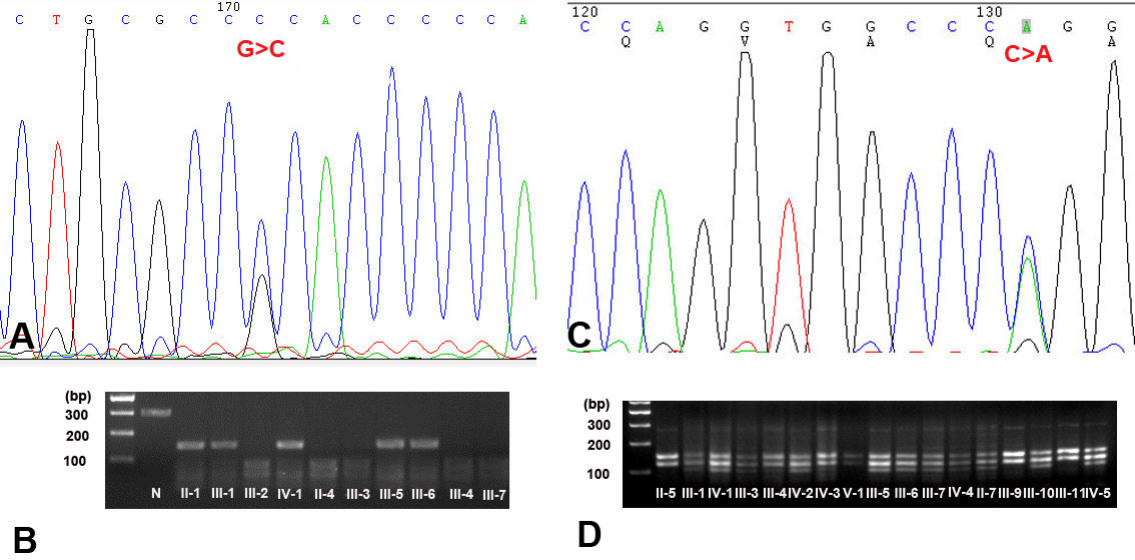
DNA sequence chromatograms and restriction fragment length analysis on the two mutations detected in this study. **A**: Heterozygote sequence (sense strand) shows a G/C transversion in codon 169 that changed alanine (GCC) to the proline (CCC) detected in family 83. **B**: c.505G>C abolished an AciI restriction site that cosegregated with the affected individuals (37 bp, 62 bp, 36 bp, and 155 bp), but not with unaffected individuals and normal controls (37 bp, 62 bp, 36 bp, 65 bp, and 90 bp). **C**: Sequence presentation of the heterozygous a C/A transversion in codon 347 that changed proline (CCG) to glutamine (CAG). **D**: c.1040C>A created a StuI restriction site that cosegregated with the affected individuals (149 bp, 124 bp, 104 bp, and 45 bp), but not with unaffected individuals (149 bp and 124 bp).

After sequencing the *RHO* gene, we identified one novel heterozygous mutation c.505G>C (p.A169P) in family 83 and one recurrent mutation c.1040C>A (p.P347Q) in family 112 (Figure 3). Using restriction fragment length polymorphism analysis, the two mutations cosegregated with the RP phenotype, respectively (Figure 3), and the novel missense mutation was not detected in 100 normal controls.

### Bioinformatics analysis

**Figure 4:**
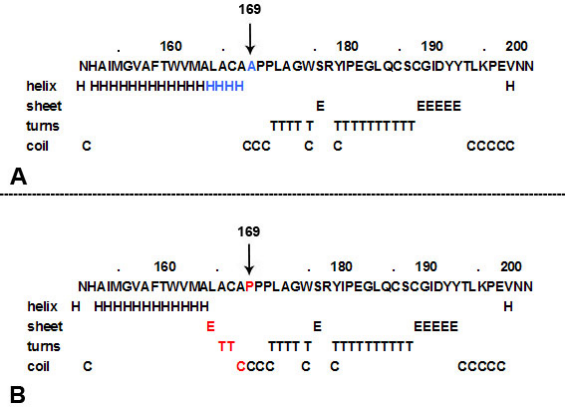
The effect of p.A169P on secondary structure of *RHO* using the GOR method [16]. **A**: The secondary structure of wild-type RHO around site A169 (in blue). **B**: The secondary structure of mutant p.A169P (in red) of *RHO* of the corresponding region, which the normal long consecutive α-helixes were shorten.

Using the GOR method, the results for secondary structure prediction suggested that the mutant *RHO* 169P replaced four helixes “H” with one β sheet “E,” two turns “T,” and one coil “C” between position 165 and 169 (Figure 4). These changes shorten the long consecutive α-helixes in the fourth transmembrane domain (residue 153–173). According to PolyPhen program analysis, p.A169P is predicted to be possibly damaging.

## Discussion

In this study, we mapped two Chinese adRP families to the *RHO* locus and identified one novel missense mutation and one recurrent mutation, respectively. The two mutations cosegregated with the phenotypes of the two pedigrees, respectively.

**Figure 5:**
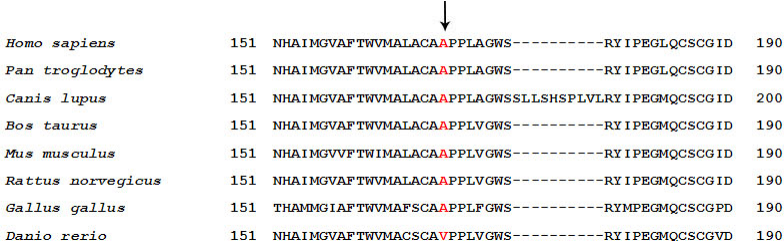
Sequence alignment portion of the fourth transmembrane domain spanning the novel missense mutation p.A169P of human *RHO* with other species.

The novel missense mutation p.A169P was identified in family 83. The Ala 169 residue is located in the fourth transmembrane domain of the rhodopsin and is relatively highly conserved (Figure 5). The result of the GOR analysis suggested that p.A169P led to significant secondary structure changes between residue 165 and 169, which may interfere with the correct folding of the transmembrane domain (Figure 4). This would classify the p.A169P within class II according to Mendes and colleagues [15]. The p.A169P substitution leads to a generalized rod-cone dystrophy phenotype in most patients; however, a variety of clinical expression was observed in the patients of family 83. Interfamilial phenotype differences have been reported in other RHO mutations [18,19]. This finding suggests that other factors (environmental or genetic) are involved in the expression of the disease.

The recurrent mutation p.P347Q was identified in family 112. Codon 347 at the C-terminus of rhodopsin is a mutational hot spot with six disease-causing sequence variations (P → S/A/R/Q/L/T) identified [20,21]. In our review of the literature, the most frequent mutations in Chinese patients with RP are in codon 347, in which p.P347L had been reported in several families or patients and p.P347S was detected in one family (Table 3). In contrast, mutations (p.P23H or P23L), which are most frequent in the American RP population [21], have not been found in Chinese patients with RP. Mutations involving codon 347 are class I mutations, which fold correctly but affect the post-Golgi trafficking of rhodopsin and impair its normal targeting to the photoreceptor outer segment [15]. Clinically, mutations involving codon 347 have been shown to be associated with the early onset and severe form of the disease [22]. This was consistent with the observations in the patients of family 112. Patients of this family had night blindness in early childhood, and visual impairment progressed with age. Two patients of this family were also diagnosed with angle closure glaucoma. Chan et al. described one Chinese patient carrying mutation p.P347L who had angle closure glaucoma [23]. It is unclear whether the glaucoma phenotype is specifically related to the rhodopsin mutation in codon 347.

**Table 3:** Summary of RHO mutations reported in Chinese patients with retinitis pigmentosa

Mutation	Onset age	Phenotype description	Glaucoma	Family or sporadic	Reference
p.E341X	23	Mild	NO	F	[24]
p.F52Y	EC	Severe	NO	F	[25]
p.V104F	NA	NA	NO	S	[22]
p.A169P	10	Variety of clinical expression	NO	F	Current Study
p.P347L	13–17	Severe	YES*	S/F/F/S/F/S	[22,23,26-29]
p.P171L	7	Severe	YES	F	[30]
p.S176F	EC	NA	NO	F	[29]
p.D190Y	NA	Severe	NO	S	[29]
p.V210F	4	Severe	NO	S	[29]
p.I256del	10	NA	NO	F	[29]
p.K311E	NA	NA	NO	S	[22]
c.980delC(p.P327fs)	30	Mild	NO	F	[23]
p.Q344R	16	Severe	NO	F/AR	[31]
p.P347S	7	Severe	NO	F	[30]
p.P347Q	5	Severe	YES	F	Current Study

Abbreviations: EC, early childhood; NA, not available; F, family; S, sporadic; AR, autosomal recessive; *, [23].

In RP, the severity of the disease seems to correlate with the localization of the *RHO* mutations. Patients in family 112 carrying the mutation p.P347Q presented with a more severe phenotype than the patients of family 83, who harbored mutation p.A169P.

In conclusion, we identified two mutations of the *RHO* gene in two Chinese families with adRP. Our findings further suggest codon 347 is the mutation hotspot of the *RHO*.

## Appendix 1. Markers used in the known ADRP genotyping.

To access the data, click or select the words “Appendix 1.” This will initiate the download of a compressed (pdf) archive that contains the file.
